# Sick Child Feeding Practice and Associated Factors among Mothers of Children Less Than 24 Months Old, in Burayu Town, Ethiopia

**DOI:** 10.1155/2019/3293516

**Published:** 2019-12-19

**Authors:** Nega Degefa, Hiwot Tadesse, Fekadu Aga, Tomas Yeheyis

**Affiliations:** ^1^Department of Nursing, College of Medicine and Health Science, Arba Minch University, Arba Minch, Ethiopia; ^2^Department of Nursing, School of Nursing and Midwifery, College of Medicine and Allied Health Sciences Addis Ababa University, Addis Ababa, Ethiopia

## Abstract

**Background:**

Growing evidence suggests that inadequate intake, poor caring practices, and disease process were some of the immediate and major causes of undernutrition in children. This points out that infant and young child feeding were the basic grounds to improve child survival and promote healthy growth and development. The first two years of a child's life are particularly important, as optimal nutrition during this period lowers morbidity and mortality, reduces the risk of chronic disease, and enhances the chances of better development. The study was aimed to assess sick infant and young child feeding practice and associated factors among mothers of children aged less than 24 months old in the Burayu town Oromia, Ethiopia.

**Methods:**

Institutional based cross-sectional study design was utilized. The study was conducted from April-May, 2015 among 362 mother–child pair attending the maternal and childcare (MCH) units of the two public health facilities in the Burayu town. Bivariate and multivariable analysis was done to test the relationship between the explanatory and outcome variables and the odds ratio with 95% confidence interval and the *p*-value was used to ascertain statistical significance.

**Result:**

More than half (53.6%) of all mothers fed their child more frequently at the time of illness than at a time of health. The mean age of respondents was 25.41 ± 3.56 and ranged from 15−30 years. Nearly three out of five (60.8%) of the respondents attended no formal education. A mother who had got counseling on sick child feeding were nearly three times more likely to feed their child appropriately than their counterparts (AOR: 2.95; 95% CI; 1.78, 4.91). Mothers who were housewives were 55% times less likely to feed their sick child appropriately than those who were working (AOR: 0.45; 95% CI; 0.26, 0.79). Those mothers who have a child aged less than 6 months were 88% less likely to practice appropriate sick child feeding than those who have a child aged more than 6 months (AOR: 0.22; 95% CI; 0.12,0.40).

**Conclusion:**

Respondents who do not receive counseling on infant and young child feeding have poor sick child feeding practice. Working mother had owned better practices of feeding child particularly at the time of illness. Infants below the age of 6 months deserve more concern in providing frequent breastfeeds at the time of illness.

## 1. Background

Adequate nutrition during infancy and early childhood is the fundamental to the development of each child's full human potential [1]. Optimal infant and young child feeding (IYCF) is found to be essential for child growth. The time during pregnancy and a child's first two years of life is considered a “critical window of opportunity” for the prevention of growth failure [2]. After birth, a child's capacity to achieve the standards in growth will be determined by the sufficiency of dietary intake (which depends on infant and young child feeding and care practices and food security), as well as exposure to diseases [3].

The World health organization (WHO) and the United Nations International Children's Emergency Fund (UNICEF) recommended early initiation of breastfeeding within one hour of birth; exclusive breastfeeding for the first 6 months of life and the introduction of nutritionally adequate and safe complementary foods at 6 months together with continued breastfeeding up to two years of age or beyond [1, 4–6].

Despite the recommendation of the WHO, poor infant feeding practices which are pooled with high rates of infectious diseases all through the first two years of life are the principal contiguous causes of malnutrition. Therefore, it is indispensable to ensure that caregivers are provided with appropriate guidance regarding the optimal feeding of infants and young children [1]. The latest Anthropometric data from low-income countries suggests that the levels of undernutrition increase obviously from 18−24 months to 3 of age [2].

Children's nutritional status can decline rapidly during/after common childhood illness if the additional nutrient requirements associated with the illness/convalescence are not properly met and the nutrients are averted from growth and development towards building the immune response. Children's poor appetite induced by illness can contribute to preserving the vicious cycle of infection and stunting [7–10].

Existing evidence suggests that, increasing fluid intake during illness, including more frequent breastfeeding and encouraging the child to eat soft, varied, appetizing, and their favorite foods. After the illness, give food more often than usual and encourage the child to eat more [11]. Even though appetite may be reduced, continued feeding of complementary food is acclaimed to preserve nutrient consumption and improve recovery [12]. After the illness, the child needs better nutrient intake to make up for nutrient losses during sickness and allow for catch-up growth. Extra food is required until the child has regained any weight loss and is growing well again.

Furthermore, appropriate infant and young child feeding during and after the illness is part of the Global Strategy for the Integrated Management of Childhood Illnesses and essential nutrition actions promoting maternal newborn, infant and young child nutrition and health [13, 14].

Regardless of the well-recognized advantages of breastfeeding worldwide, performance on recommended policies and programs for breastfeeding is poor. No country highly adhere to all of the indicators, proving that significant progress on all fronts is looked-for. Unluckily, countries are not adequately protecting, promoting, or supporting breastfeeding [15].

Despite the overwhelming evidence on the benefits of exclusive breastfeeding, only about one in three African babies under six months are exclusively breastfed, due to the lack of understanding of optimal feeding practices and the lack of support from health care providers, community members, and families. Babies who are not exclusively breastfed in the early months have a higher risk of death, especially from infection [16].

Breastfeeding is nearly universal in Ethiopia and half of the children born in the three years before the survey are breastfed for about 25 months. More than half (52%) of the children less than 6 months old are exclusively breastfed in Ethiopia [17]. The feeding practices of only 7% of children in Ethiopia age 6−23 months meet the minimum requirement with respect to all the three IYCF practices (breastfeeding status, number of food groups, and times they were fed during the day or night before the survey). Fourteen percent of children had a sufficiently varied diet in which they had been given foods from the appropriate number of food groups, and 45% had been fed the least possible number of times appropriate for their age [17].

Despite a few local studies conducted in different places in the country which explores feeding practice generally [18–20]: there is a shortage of evidence in assessing sick child feeding practice and factors associated with. Hence, this study was aimed to assess sick child feeding practice and associated factors among mothers of under 24-month-old child in Burayu town.

## 2. Methods and Materials

### 2.1. Study Design and Area

A facility-based cross-sectional study was conducted from April to May 15, 2015. The study was conducted at public health facilities found in Burayu town which is located at 12 km west to Addis Ababa. The total population of the town was estimated to be 63,873 of whom 31,504 are men and 32369 women [21]. There are 2 health centers and 2 health posts owned by the government, and 40 clinics owned by private organizations. The majority of the town's population receive service from government-owned health facilities.

### 2.2. Inclusion and Exclusion Criteria

All mothers who visited public health centers in Burayu town during the data collection period were included in the study while those mothers who had a serious illness or seriously ill child were excluded from the study.

### 2.3. Sample Size, Study Population and Sampling Procedure

The sample size was calculated using a single population proportion formula by considering the following assumptions: the proportion of mothers who properly practice sick baby feeding was (45.0%) from a study by Agumasie et al. in Ethiopia [22]. Confidence interval of 95%, 5% margin of error and 10% none-response. A final sample size of 418 mother-child pair was obtained.

Respondents of the study were taken, from each of the two public health centers in Burayu town depending on their predetermined client flow rate. To enroll respondents into the study: first, the average number of mothers who visited maternal and child health clinic daily at the two health centers were identified by referring client registration book for the last two months prior to data collection. Afterward, participants were included proportionally by considering a possible number of client that can be expected in each health center during the data collection period. Systematic random sampling method was used to select study participants. The average daily client flow rate for the first health center was 16 client and 18 for the second which gives an average monthly client flow rate of 480 and 540 respectively. So the sample was taken from each health center proportional to the expected number of client flow rate so, we took 170 and 192 respondent from the first and second health center respectively.

### 2.4. Operational Definition and Measurement

Dependent (outcome) variable: sick child feeding practice (*good/poor*)

*Sick child-* refers to an infant or young child who had either of the common childhood illness like pneumonia or diarrhea and seeks treatment.

*Sick child-feeding practice –* refers to routines of feeding a child at the time of illness. To assess these mothers were asked a question on how frequent they fed their child at the time of illness (the correct answer was more than 2-3 meals per day for those aged 6–8 month, more than 3-4 meals per day for those 9–23 months). And for those who were exclusively breastfed mothers who fed more than the normal frequency (8–12 feeds per day) had *good sick child-feeding practice* whereas those mothers who gave the usual amount of liquids and those giving somewhat less amount and frequency of liquids than usual or withholding feeding were considered as having *poor sick baby-feeding practice* [11].

### 2.5. Data Collection Tool and Procedure

Data were collected from mothers of under 24-month-old child by using structured and interviewer-administered questionnaire. The questionnaire was initially prepared in the English language and then translated to the local language (Afan Oromo) and the responses were translated back to English to check for consistency. Four diploma clinical nurse who can fluently speaks the local language and be available throughout the data collection time undertake the face to face interview and one health officer supervised the overall process on a daily basis.

### 2.6. Data Quality Control

The collected was checked manually for completeness, cleaned and double entered by using Epidata version 3.1. A pretest was done on 5% of the sample and the result was used to adjust the content and approach of the questionnaire. Two days training was given both for data collectors and supervisor on the whole process of data collection. The supervisor controls the completeness of the questionnaire and consistency of the data and communicates with the principal investigator in cases of difficulties.

### 2.7. Data Management and Analysis

The data were analyzed using SPSS version 20. Descriptive statistics like frequency distribution, percentage, and means were used to define respondents in relation to pertinent variables and presented using tables and graphs. Variables which showed association with the dependent variable in the bivariate analysis at alpha <0.25 were entered into the multivariable logistic regression model. Adjusted Odds ratios (OR) with corresponding 95% confidence interval was estimated and *p*-value less than 0.05 were used to identify variables that had a statistically significant association with mothers sick child feeding practice in the final model. Multicollinearity among independent variables was tested by computing variance inflation factor and looking standard error. Hence (VIF > 10) and (SE > 2) was suggestive of collinearity. Model fitness was tested by Hosmer-Lemeshow goodness of fit test.

### 2.8. Ethical Clearance

Ethical approval was obtained from Addis Ababa University College Medicine and allied health Science Ethical Review Board. Respondents were given information regarding the purpose of the study and assured that all information they provide will be confidential. All of the respondents signed on the written informed consent form prior to participation.

## 3. Result

### 3.1. Sociodemographic Characteristics of the Mother and Child

A total of 362 mother-child pair participated in the study with a response rate of 87%. The mean age of respondents was 25.41(±SD 3.56) and ranges from 15–30 years. Nearly half (48.1%) of all respondents had Oromo ethnic group and forty-four percent of the mothers were orthodox religion followers. Almost all (97%) of the respondents were married. Majority of the respondents (88.4%) had attended formal education (Table 1).

### 3.2. Health Care Service Utilization and Obstetrics Related Characteristics of the Mothers

Almost all of the mothers had antenatal care follow up however only less than half (47%) of them had got counseling on infant and young child feeding. About 35% of mothers used bottle for child feeding. Nearly 94% of mothers gave birth for an index child at health institution and assisted by a health professional (Table 2).

### 3.3. Sick Child Feeding Practice of Mothers

**Figure 1:**
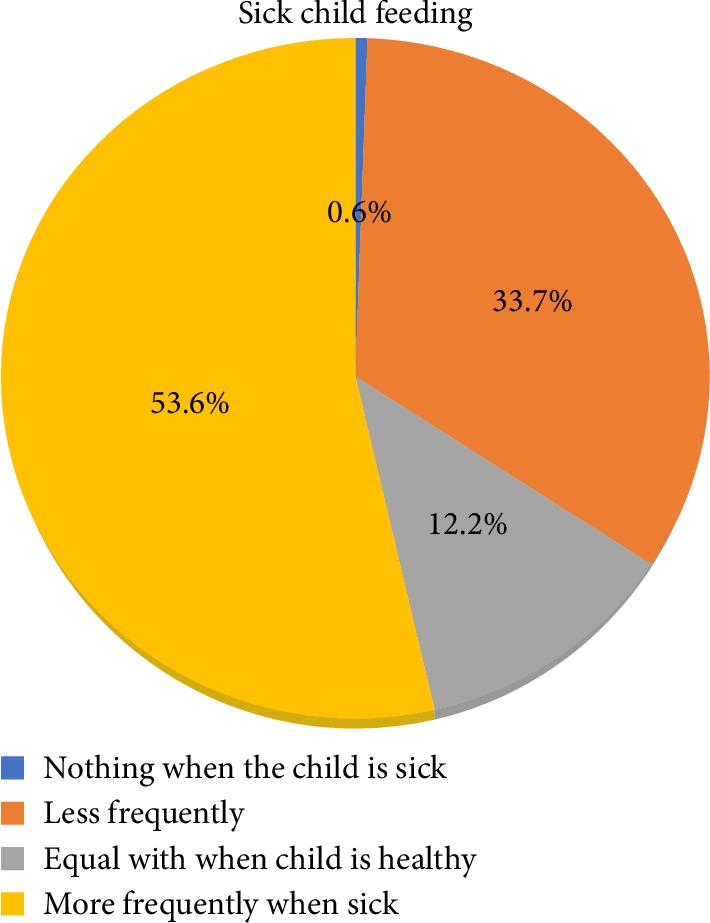
Sick child feeding practice of mothers in Burayu town, Oromia, Ethiopia, 2015.

The current study showed that nearly fifty-four percent of mothers had a good practice of sick child feeding. Hence those mothers feed their sick child more frequently at a time of illness than when they were healthy (Figure 1).

### 3.4. Factors Associated with a Sick Infant and Young Child Feeding Practice of Mothers

As shown on the table below, mother's occupation, access to counseling of infant and young child feeding and age of the child were factors that have a statistically significant association with mother's sick child feeding practice. Hence, a mother who had got counseling on sick child feeding were almost three times more likely to feed their child appropriately than those who did not get counseling (AOR: 2.95; 95% CI; 1.78, 4.91). Likewise, housewife mothers were 55% times less likely to feed their sick child appropriately than those who worked outside the home (AOR: 0.45; 95% CI; 0.26, 0.79). Those mothers who have a child aged less than 6 months were 88% times less likely to provide more frequent feeds to their sick child than those who have a child aged greater than 6 months (AOR: 0.22; 95% CI; 0.12,0.40) (Table 3).

## 4. Discussion

The current study showed that more than half (53.6%) of mothers had an appropriate sick infant and young child feeding practice which means, the mentioned proportion of mother provide their child with breast milk or soft and appetizing complementary diet more frequently at the time of illness than when they were normal. A study by Semahagn et al. in Ethiopia showed that 45.0% of mothers provide to their child more frequent feeds at the time of illness [22]. Which is slightly lower than the finding in the current study. The difference may be attributed to the variation in the time of the two studies. A nearly consistent finding was reported on a study by Giri et al. in which 51.5% of the mothers increases the frequency of feeding to their sick child [23]. The finding of a study by Dongre et al. in a tribal district of Maharashtra, India that investigated household practices for the sick child also showed, that, the status of some desired household practices such as frequent feeding and giving extra fluid to drink during episodes of illness was poor [24].

The finding of our study revealed that 33.7% of mothers provide their children with less frequent feeds during illness almost similar finding was reported on study in Tanzania which revealed that, some (35%) of mothers offered their children less amount of breast milk or nonbreast milk liquids (24%) because of the child's refusal to feed during illness [25]. Consistent to this finding, a study by Benakappa et al. that evaluates beliefs of caregivers regarding diet during childhood illness; showed that a child must be fed less during illness [26] and 21% of mothers in a study by Giri et al. believed that breastfeeding should be decreased during illness [23].

In the current study, working mothers were more likely to continue feeding and gave more frequent feeds than housewives consistently, the finding of a study by Dongre et al. which stated that working mothers more commonly continued breastfeeding and gave more frequent feed than their counterpart at the time of illness [24]. This may be attributed to the difference in the level of education and understanding of working mothers as compared to housewives. Even though working mothers spent most of their day out of home, they strongly adhere to the providers counseling and look after their baby at the time of illness and feed them properly than a housewife.

The finding of our study showed that only 0.6% mothers stopped feeding the baby at the time of illness while a study by Gupta et al. that investigates the first action of mothers for a child with diarrhea reported 7.8% mothers to stop feeds that, includes breast-feeds and other fluids [27]. The discrepancies may possibly be supported by the difference in time between the two studies, the social and cultural variation among respondents.

In the present study mothers of younger infants were less likely to breastfeed more frequently than mothers of older children who gave more complementary foods during and after an illness. This may be attributed to the poor appetite of infants induced by illness and dependency on only breast milk among infants who didn't start complementary foods. However, the mother's of younger children those who start complementary food can give different types and appetizing complementary food items in addition to breastmilk.

The primary strength of this study is the application of a proper model for analysis and sampling which is representative of the target population. The study also had limitation like the inability to establishing a causal relationship between the explanatory and outcome variable due to the cross-sectional nature of the study. Besides we fail to observe the practice of mothers while they provide feeds exactly at the time of illness despite their response of how frequent they feed their child both when they are healthy and sick. These drawbacks will deter the implication of our finding, so the further study should be carried out to explore the detailed practice by observing the normal meal frequency given to the baby at the time of illness.

## 5. Conclusion and Recommendation

There is increasing dissemination of information on infant and young child feeding (IYCF) behaviors and practices in Ethiopia. However, still, there is limited information about infant and young child feeding at the time of illnesses. Evidence showed that implementation of IYCF behaviors and practices during common childhood illnesses are far from optimal. In general, sick children continue to be breastfed. However, few are breastfed more frequently to compensate for the additional fluid and nutrient requirements which are associated with the illnesses, while a significant proportion of children were breastfed less frequently than usual.

Restriction or withdrawal of breastfeeding or complementary foods during illness is common among respondents, as a result of children's loss of appetite (supposed or actual), and poor awareness of caregivers' about the feeding needs of sick children. Consequently, many sick children were fed less frequently during illness. It would be better if the IYCF behaviors and practices could be strengthened specifically focusing on feeding during and after a common childhood illness.

## Figures and Tables

**Table 1:** Sociodemographic characteristic of respondents (*n* = 362) visiting MCH units of health centers in Burayu town, Oromia, Ethiopia, 2015.

Variables	Category	Frequency (*n*)	Percent (%)
Mother's age	15–19	3	0.80
	20–24	151	41.7
	25–29	161	44.5
	30–34	42	11.6
	≥35	5	1.40

Ethnicity	Oromo	174	48.0
	Amhara	42	11.6
	Tigre	18	5.00
	Gurage	81	22.4
	Wolayita	34	9.40
	Other	13	3.60

Mother's religion	Orthodox	160	44.2
	Muslim	90	29.3
	Protestant	106	59.4
	Other	6	1.70

Marital status	Married	351	97.0
	Single	8	2.20
	Divorced	2	0.60
	Widowed	1	0.30

Mother's occupation	Housewife	266	73.5
	Working outside	96	26.5

Mothers education	Formal education	320	88.4
	No formal education	42	11.6

Monthly income (ETB)^∗^	≤1000	147	40.6
	1001–2000	114	31.5
	≥2001	101	27.9

^∗^Ethiopian birr.

**Table 2:** Health care service utilization and obstetrics related characteristics of the mothers of under 24-month-old child (*n* = 362) visiting the MCH unit of health centers in Burayu town, Oromia, Ethiopia, 2015.

Variables	Category	Frequency (*n*)	Percent (%)
Parity	1-2	147	40.6
	3-4	114	31.5
	5 & above	101	27.9

ANC follow up	Yes	353	97.5
	No	9	2.5

Receive counseling on IYCF	Yes	169	46.7
	No	193	53.3

Source of information on sick baby feeding	Health professionals	266	62.4
	Health extension workers	24	6.6
	Mass media	43	11.9
	Other	29	9.1

Place of delivery	Home	20	5.5
	Health institution	342	94.5

Bottle feeding	Yes	127	35.1
	No	235	64.9

Birth attendant	TBA	7	1.9
	Health professional	343	94.7
	Relatives	12	3.3

Accesses to media	Radio	278	76.8
	Television	248	68.5
	Magazine/books/news paper	54	14.9

**Table 3:** Bivariate and multivariable analysis of sick child feeding practice and associated factors among mothers of under 24-month-old child visiting the MCH unit of health centers in Burayu town, Oromia, Ethiopia, 2015.

Variables	Sick child feeding practice		95% CI	
	Good	Poor	COR	AOR
*Occupation of mother*				
Housewives	158 (59.4)	108 (40.6)	2.14 (1.32,3.43)	0.45 (0.26,0.79)^∗^
Working mothers	39 (40.6)	57 (59.4)	1	1

*Receive counseling on IYCF*				
Yes	113 (66.9)	56 (33.1)	2.61 (4.01, 11.70)	2.95 (1.78,4.90)^∗^
No	84 (43.5)	109 (56.5)	1	1

*Child age*				
<6 months	42 (28.7)	91 (71.3)	0.23 (0.13,0.39)	0.22 (0.12,0.40)^∗^
6–12 months	71 (68.9)	32 (31.1)	1	1
>12	80 (63.5)	46 (36.5)	1	1

*Monthly income*				
<1000	66 (44.9)	81 (55.1)	0.87 (0.68, 1.128)	
1000–2000	46 (40.4)	68 (59.6)	1	
>2000	53 (52.5)	48 (47.5)	1	

*Mothers education*				
Formal education	172 (53.8)	148 (46.3)	0.79 (0.38, 1.44)	
No formal education	25 (61.0)	17 (39.0)	1.	

^∗^ *P* < 0.05. IYCF- infant and young child feeding.

## Data Availability

You can have the data set used and/or analyzed during the current study from the following authors (Hiwot Tadesse, Nega Degefa) on a reasonable request.

## Abbreviations

ANC: Antenatal care
PNC: Postnatal care
EDHS: Ethiopian demographic and health survey
WHO: World Health Organization
AOR: Adjusted odds ratio
CI: Confidence interval
IYCF: Infant and young child feeding
UNICEF: United Nations international children's emergency fund.
